# Fluorescence and Cytotoxicity of Cadmium Sulfide Quantum Dots Stabilized on Clay Nanotubes

**DOI:** 10.3390/nano8060391

**Published:** 2018-05-31

**Authors:** Anna V. Stavitskaya, Andrei A. Novikov, Mikhail S. Kotelev, Dmitry S. Kopitsyn, Elvira V. Rozhina, Ilnur R. Ishmukhametov, Rawil F. Fakhrullin, Evgenii V. Ivanov, Yuri M. Lvov, Vladimir A. Vinokurov

**Affiliations:** 1 Functional Aluminosilicate Nanomaterials Lab, Gubkin University, Moscow 119991, Russia; stavitsko@mail.ru (A.V.S.); kain@inbox.ru (M.S.K.); kopicin.d@inbox.ru (D.S.K.); ivanov166@list.ru (E.V.I.); vinok_ac@mail.ru (V.A.V.); 2 Bionanotechnology Lab, Institute of Fundamental Medicine and Biology, Kazan Federal University, Kazan, Republic of Tatarstan, Russian Federation, 420008; rozhinaelvira@gmail.com (E.V.R.); sal.ilnur@gmail.com (I.R.I.); kazanbio@gmail.com (R.F.F.); 3 Institute for Micromanufacturing, Louisiana Tech University, Ruston, LA 71272, USA

**Keywords:** bioimaging, nanoarchitectures, halloysite, intracellular labeling

## Abstract

Quantum dots (QD) are widely used for cellular labeling due to enhanced brightness, resistance to photobleaching, and multicolor light emissions. CdS and Cd_x_Zn_1−x_S nanoparticles with sizes of 6–8 nm were synthesized via a ligand assisted technique inside and outside of 50 nm diameter halloysite clay nanotubes (QD were immobilized on the tube’s surface). The halloysite–QD composites were tested by labeling human skin fibroblasts and prostate cancer cells. In human cell cultures, halloysite–QD systems were internalized by living cells, and demonstrated intense and stable fluorescence combined with pronounced nanotube light scattering. The best signal stability was observed for QD that were synthesized externally on the amino-grafted halloysite. The best cell viability was observed for Cd_x_Zn_1−x_S QD immobilized onto the azine-grafted halloysite. The possibility to use QD clay nanotube core-shell nanoarchitectures for the intracellular labeling was demonstrated. A pronounced scattering and fluorescence by halloysite–QD systems allows for their promising usage as markers for biomedical applications.

## 1. Introduction

Nanomaterials show great promise when it comes to targeted drug delivery, diagnostics, and controlled-release drug treatment. In recent years, formulations based on halloysite clay nanotubes have attracted attention in biology and medicine as a drug delivery vehicle [1,2,3,4,5,6], in implants and tissue engineering [7], and in chemical- and bio-sensing [8,9]. Halloysite nanotubes can be employed as encapsulation containers due to their tubular structure, mesoporous 10–15 nm diameter lumen, and site-dependent chemistry with positively (Al_2_O_3_) and negatively (SiO_2_) charged inner and outer tube surfaces [10,11,12,13].

Halloysite has been proposed as a template for synthesis and stabilization of various nanoparticles following a core–shell nanoarchitecture strategy. Halloysite–metal nanoparticle composites have been employed in heterogeneous catalysis [14,15]. Antibacterial Ag [16,17], as well as plasmonic Au nanoparticles [18], were synthesized into and onto halloysite tubes to form new nanosystems with enhanced biological activity. Metal chalcogenide halloysite-based formulations have already been tested in photocatalysis and bioimaging [19,20]. Metal chalcogenide quantum dots (QD) are semiconductor nanoparticles with a size of up to 10 nm that emit light with a wavelength that can be finely tuned from ultraviolet to infrared, depending on sizes, structure, and composition; among them, cadmium containing QDs are one of the most useable [21]. QDs are favorable for intracellular labeling because of the easily tunable light emission, wide absorption band, high resistance to photobleaching, and possibilities of surface modification by conjugation with proteins [22].

One of the main problems with nanomaterial applications in living organisms is the toxicity of such tiny objects. For example, QD for bioimaging are often synthesized using complicated stabilization techniques to make them less cytotoxic and better dispersible in water, and this is especially important for cadmium-containing QDs [23]. Halloysite clay encapsulation could help dispersing the quantum dots in water, as well as decrease the toxicity of QD via the tube surface immobilization and decreasing amounts of free CdSe or CdS. These natural alumosilicate clay rolled structures, named halloysite, are known to be biocompatible [24]. One of the first reports on halloysite exposition for HeLa and MCF-7 mammal cell lines demonstrated that halloysite has low toxicity at concentrations up to 100 μg/mL. Although halloysite can be easily taken up by the cultured cells, its toxicity was reported to be very low, both for cell cultures and in vivo for animals [25]. Even at higher concentrations (up to 1500 μg/mL), halloysite nanotubes conjugated with noble metal nanoparticles, which have low toxicity for plants, as was reported for radish seeds [26].

Here, we report that site-selective immobilization of cadmium-containing QDs on halloysite nanotubes opens the way to obtain new fluorescent materials with broad emission spectra, good stability, and low in vitro toxicity. To reduce the cadmium content, a solid solution of cadmium-zinc sulfide was also used as fluorescent nanoparticle shells on core clay nanotubes.

## 2. Materials and Methods

Halloysite nanotubes (HNT), (3-Aminopropyl)triethoxysilane (APTES), furfural, hydrazine hydrate, cadmium nitrate tetrahydrate (Cd(NO_3_)_2_·4H_2_O), zinc nitrate hexahydrate (Zn(NO_3_)_2_·6H_2_O), thioacetamide (TAA), ethylenediaminetetraacetic acid (EDTA), ammonium hydroxide solution (NH_4_OH), and ethanol 96% were all purchased from Sigma-Aldrich (Rushim, Moscow, Russia).

### 2.1. QDs Stabilization on Halloysite

Cadmium sulfide and cadmium-zinc sulfide QDs were stabilized on halloysite using the two following synthesis strategies.

**Scheme 1:**
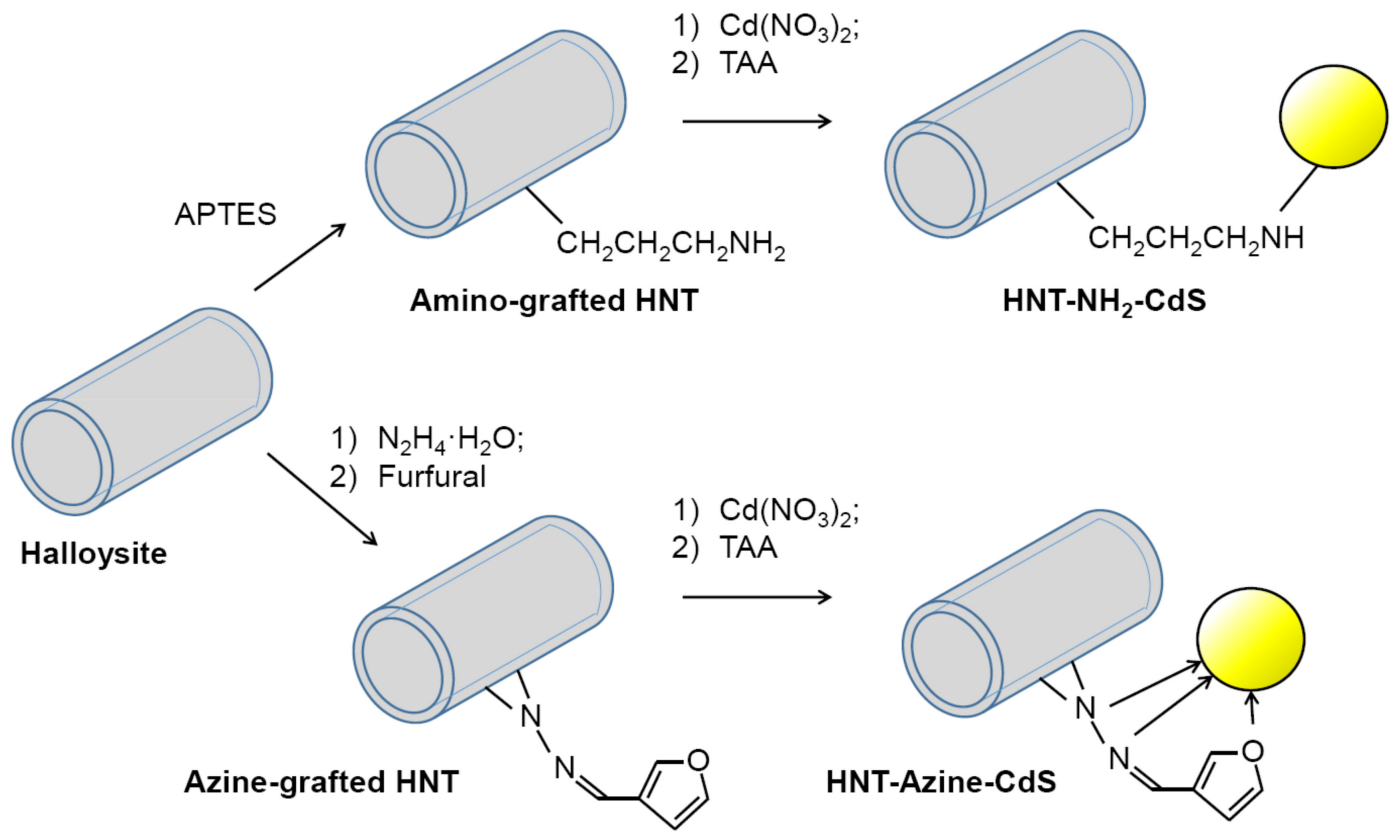
Synthesis of halloysite-CdS composites.

In first case, APTES was used as a grafting agent. The halloysite salinization was performed using 0.2 g of APTES per 1 g of halloysite dispersed in ethanol and stirred at 60 °C for 24 h (modified from [27]). After the reaction, the dispersion was washed several times with ethanol. The resulting precipitate was dried for 12 h at 60 °C. Afterwards, the resulting HNT-NH_2_ was dispersed in a Cd(NO_3_)_2_ solution and stirred for 30 min using sonication. Then a TAA solution was added in the cadmium-containing mixture, and pH was adjusted to 10 using NH_4_OH. After 5 min, the yellow precipitate was centrifuged, washed with ethanol several times, and dried at 60 °C for 24 h. The obtained sample was labeled as HNT-NH_2_-CdS (see Scheme 1).

In second case, azine produced from furfural and hydrazine hydrate was used as a ligand to form stable CdS and Cd_x_Zn_y_S QD on halloysite nanotubes. The procedure for HNT-Azine synthesis is described elsewhere [20,28]. The synthesis of CdS QDs was performed according to the same procedure described above. The obtained sample was labeled as HNT-Azine-CdS. The same HNT-Azine was used to stabilize Cd_0.7_Zn_0.3_S QD, where a solution of Cd(NO_3_)_2_ and Zn(NO_3_)_2_ with Cd:Zn molar ratio of 0.7:0.3 was taken as a metal precursor solution. The procedure of QD synthesis was the same as for HNT-NH_2_-CdS and HNT-Azine-CdS. The sample was labeled as HNT-Azine-Cd_0.7_Zn_0.3_S.

To compare the photostability of synthesized HNT-QD materials with commonly used fluorescent dyes, we prepared rhodamine 6G (R6G) and fluorescein (Fluor) dyes that were adsorbed onto the halloysite nanotubes. Dyes were adsorbed on the halloysite surfaces from concentrated ethanol solutions, using a vacuum for better loading. After soaking the halloysite in dye solution for 30 min, the ethanol was evaporated under vacuum, and obtained composites (HNT-R6G and HNT-Fluor) were washed with ethanol and dried overnight.

### 2.2. Cell Cultures

Epithelial human prostate cell line (PC-3) was obtained from the American Type Culture Collection (ATCC, Manassas, VA, USA). The cells were seeded in a sterile culture flask with a growth area of 75 cm^2^ (Corning Inc., Corning, NY, USA), and contained 12 mL of Dulbecco’s Modified Eagle’s Medium (DMEM), supplemented with 10% fetal bovine serum (PAA laboratories, Dartmouth, MA, USA), 100 IU/mL of penicillin, and 100 ng/mL of streptomycin. The cells were cultivated at 37 °C in a humidified atmosphere containing 5% CO_2_, and sub-cultured by trypsinization every three days at 80% confluency.

### 2.3. Characterization

Fluorescent materials’ morphology evaluation and elemental analysis were performed with a JEM-2100 transmission electron microscope (Jeol, Tokyo, Japan), equipped with a JED-2300 X-ray fluorescence spectrometer (Jeol, Tokyo, Japan). Size distributions of QD were estimated by measuring the diameters of electron-dense particles in transmission electron microscopy (TEM) images with ImageJ v1.50i suite (National Institutes of Health, Bethesda, MD, USA). Reflectance spectra of the synthesized materials were registered in 45°/45° geometry using a 150 W xenon arc lamp (LOT Oriel, Darmstadt, Germany) and QE65000 spectrometer (Ocean Optics, Dunedin, FL, USA). Elemental analysis was performed using an ARL™ PERFORM’X Sequential X-ray Fluorescence Spectrometer (Thermo Scientific, Waltham, MA, USA).

For the study of cells’ morphology and cytoskeleton structure, 1 × 10^5^ cells were added to the six-well plate re-suspended in medium (1 mL) on coverslips (10 mm), where the plate was previously at the bottom of each well. The cells were grown for 24 h, the HNT-QD (100 mg/mL) was added, and cells were cultured for 24 h (37 °C and 5% CO_2_). Then, the cells were washed with phosphate-buffered saline (PBS) and stained with 4′,6-diamidino-2-phenylindole (DAPI) solution (1 mg/mL) and Phalloidin Alexa Fluor^®^ 488 (Thermo Fisher Scientific Inc., Waltham, MA, USA), according to the standard protocols. The slides were observed with laser scanning microscope Carl Zeiss LSM-780 (Jena City, Germany) with 543 nm, 488 nm, and 405 nm lasers, and images were processed using ZEN Black software (Carl Zeiss MicroImaging GmbH, Göttingen, Germany). Enhanced dark-field microscopy images were obtained using an Olympus BX51 upright microscope (Tokyo, Japan) equipped with a CytoViva^®^ oil immersion dark-field condenser (Auburn, AL, USA).

### 2.4. Cell Viability

To measure the cell viability while incubated with HNT-QD, they were seeded in a sterile culture flask with growth area of 75 cm^2^ (Corning Inc., Corning, NY, USA) until the confluence reached ~80%. Cells were rinsed with PBS and then were detached from the substrate by trypsinization. Then, the cells were grown in a six-well plate for 24 h and HNT-QD were added. After 24 h of incubation in standard conditions, we assessed cell death induction using ReadyProbes^®^ Cell Viability Imaging Kit (Blue/Green) (Thermo Fisher Scientific Inc., Waltham, MA, USA) according to the standard protocol. The nuclei of all cells were analyzed with a standard DAPI filter (excitation/emission maxima: 360/460 nm), and the nuclei of dead cells with compromised plasma membranes were detected with standard FITC/GFP (green) filter set (excitation/emission maxima: 504/523 nm) on a flow cytometer FACS (BD Biosciences, San Jose, CA, USA).

## 3. Results

### 3.1. Fluorescent Materials Morphology and Composition

Halloysite nanotubes (HNT) that were used varied in length from 300 nm to 1 µm, with an average length of 600 nm, inner diameter of 15–20 nm, and their chemical formula is similar to kaolinite (Al_2_Si_2_O_5_(OH)_4_·nH_2_O). The outer surface of halloysite is negatively charged at pH above 4 and was comprised of silica [29]. Surface modification of halloysite with silane is a common method that allows nanoparticles to bind to halloysite surface and to prevent their detachment from the tubes and aggregate [30,31,32]. In the case of cadmium containing QD (CdS or Cd_0.7_Zn_0.3_S), it is preferable to have complexation agents that cover QD to prevent cadmium release. Therefore, we have chosen an organic azine as a ligand for chalcogenide nanoparticle formation. This technique makes it possible to load nanoparticles inside the lumen of halloysite nanotubes [15], preventing QD nanoparticles from aggregating.

**Figure 1:**
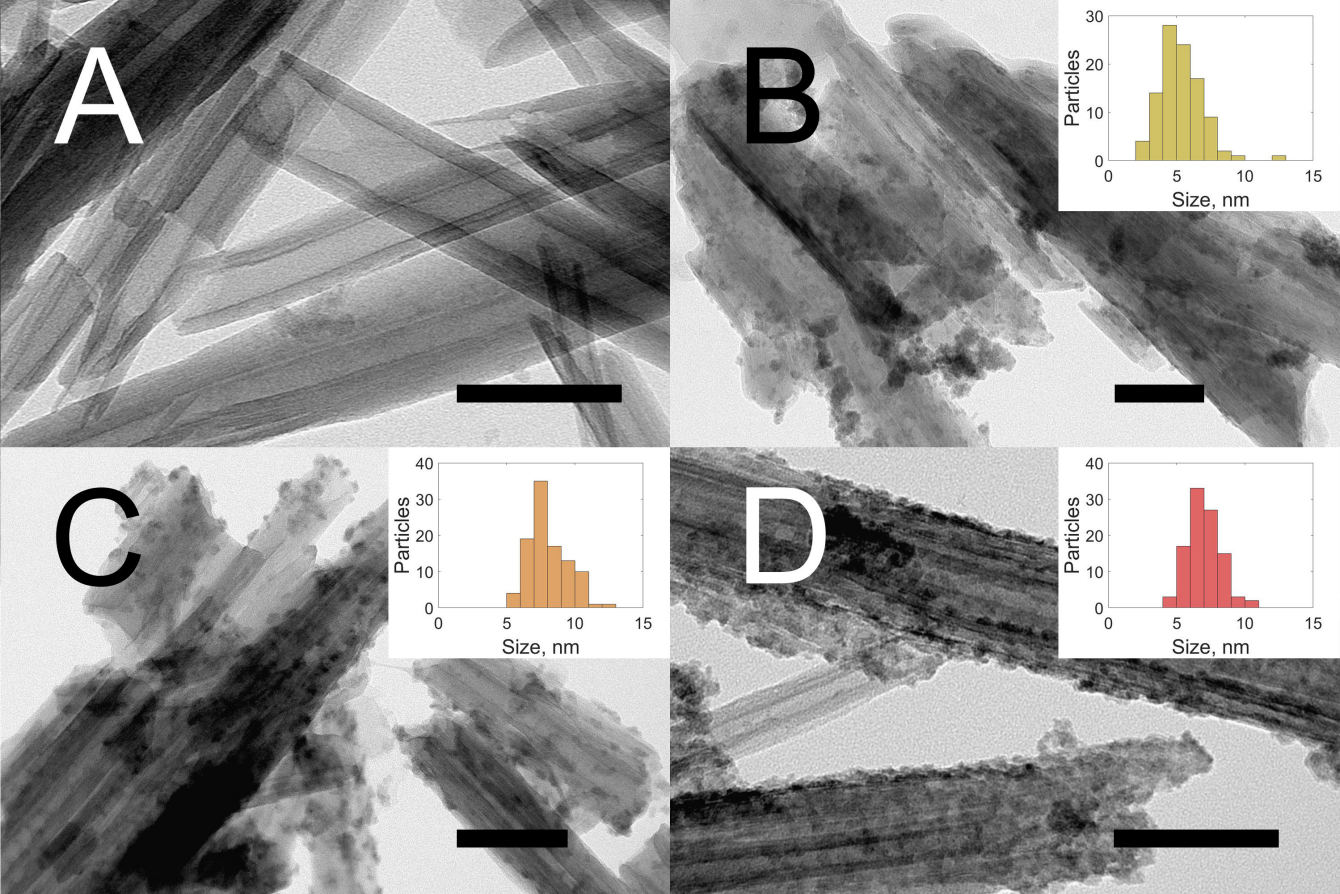
TEM images of pristine HNT (**A**); HNT-NH_2_-CdS (**B**); HNT-Azine-CdS (**C**); and HNT-Azine- Cd_0.7_Zn_0.3_S (**D**). Particle size distributions derived from measurements of 100 particles for each sample are shown in insets (**B**–**D**), (see also Table S1 in Supplementary Materials).

Figure 1 shows TEM images of halloysite nanotubes before modification (A), and after synthesis of fluorescent nanomaterials (B–D). The content of CdS in the samples, as evaluated by X-ray fluorescence elemental analysis, was 3.2, 3.5, and 2.9 wt% for cadmium sulfide quantum dots immobilized onto the amino-grafted halloysite HNT-NH_2_-CdS (Figure 1B), cadmium sulfide quantum dots immobilized onto the azine-grafted halloysite HNT-Azine-CdS (Figure 1C), and cadmium-zinc sulfide quantum dots immobilized onto the azine-grafted halloysite HNT-Azine-Cd_0.7_Zn_0.3_S (Figure 1D), respectively.

As shown above, the particle location on the tubes was different in every case. In the case of amino-grafted halloysite, the clusters were distributed all over the nanotubes. The particle size varied in a wide range from 2 to 13 nm (Figure 1B, inset). The concentration of nanoparticles in case of amino-grafted halloysite (Figure 1B) was lower than that of azine-grafted QD (Figure 1C,D). HNTs-Azine-CdS materials contained QD with better monodispersity; the majority of QD have size within 6 to 8 nm (Figure 1C,D, insets). There were no large Cd-clusters observed separate from the nanotubes. HNTs-Azine-Cd_0.7_Zn_0.3_S had densely located particles with size from 5 to 10 nm. Such a difference in the nanoparticle size distributions caused a variation in the material’s spectral properties.

**Figure 2:**
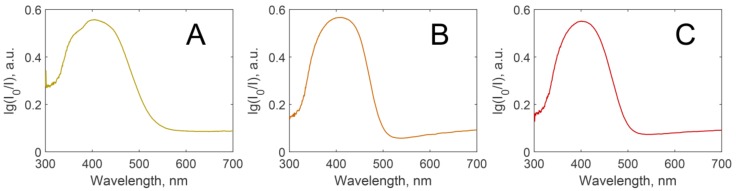
Diffusive reflectance spectra of HNT-NH_2_-CdS (**A**); HNT-Azine-CdS (**B**); and HNT-Azine-Cd_0.7_Zn_0.3_S (**C**). Spectra were registered using pristine halloysite as a reference.

The synthesized cadmium QD materials had a bright yellow color, which is associated with their strong light absorption in blue spectral range (Figure 2). The positions of absorption peaks imply that these nanomaterials might demonstrate fluorescence when excited by a laser in the 400–500 nm range.

### 3.2. Laser Scanning and Dark Field Microscopy

**Figure 3:**
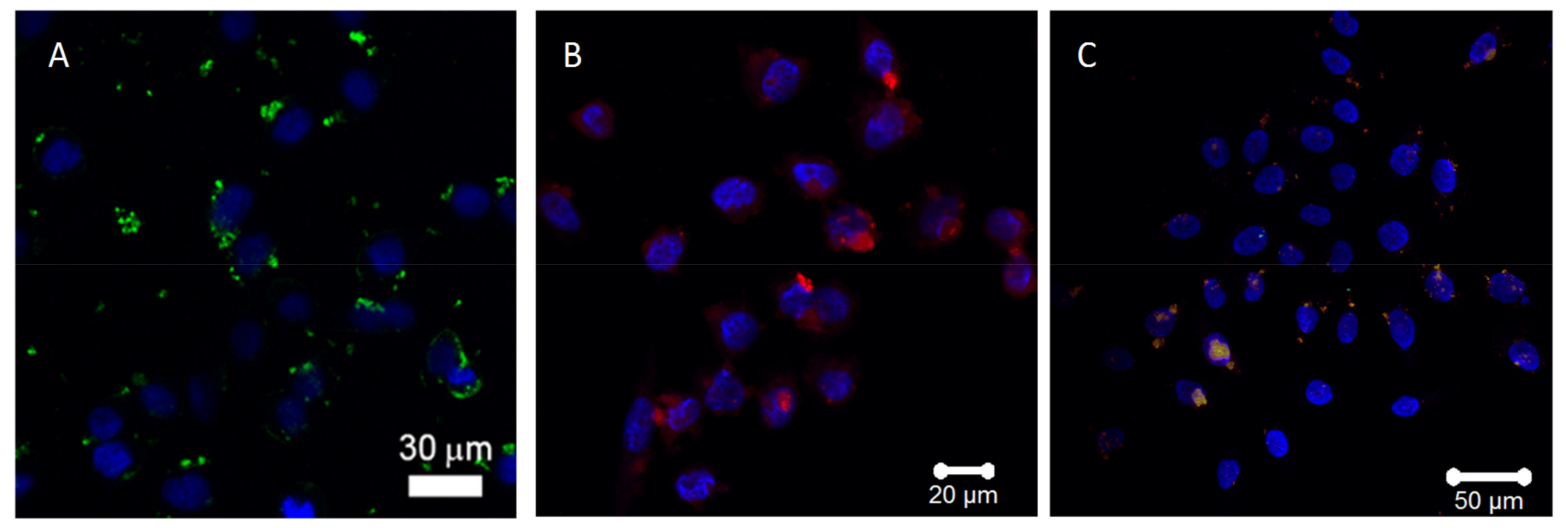
Laser scanning microscopy (LSM) images of PC-3 cells that were QD-labeled with HNTs-Azine-CdS (**A**), HNTs-NH_2_-CdS (**B**), and HNTs-Azine-Cd_0.7_Zn_0.3_S (**C**). The nuclei were stained with DAPI (blue channel, 405-nm laser), QD are shown in green (488-nm laser) and red channel (543-nm laser).

We applied the halloysite-QD composites to label human cells in vitro to demonstrate the optical effects of these materials within live cells. Figure 3 presents the laser scanning microscopy (LSM) images of different Cd-composites with QD stabilized on halloysite nanotubes. Bright and well-resolved fluorescence was observed in all cases. It can be seen that every material had different emission spectra ranging from green (HNTs-Azine-CdS), to yellow-red (HNTs-Azine-Cd_0.7_Zn_0.3_S), to red (HNT-NH_2_-CdS). Figure 3C shows the confocal microscopy image of the sample that had been stored for nine months. Cell nuclei were stained with DAPI, and were visible as large blue spots. Smaller red, yellow-red, and green spots around the nuclei correspond to HNT-QD composites, and imply that these composites are well-distributed on the cells’ surfaces or inside them. These images confirm the effective uptake of QD-modified halloysite; apparently the uptake occurs in the same way as with dextrin-coated clay nanotubes [6].

**Figure 4:**
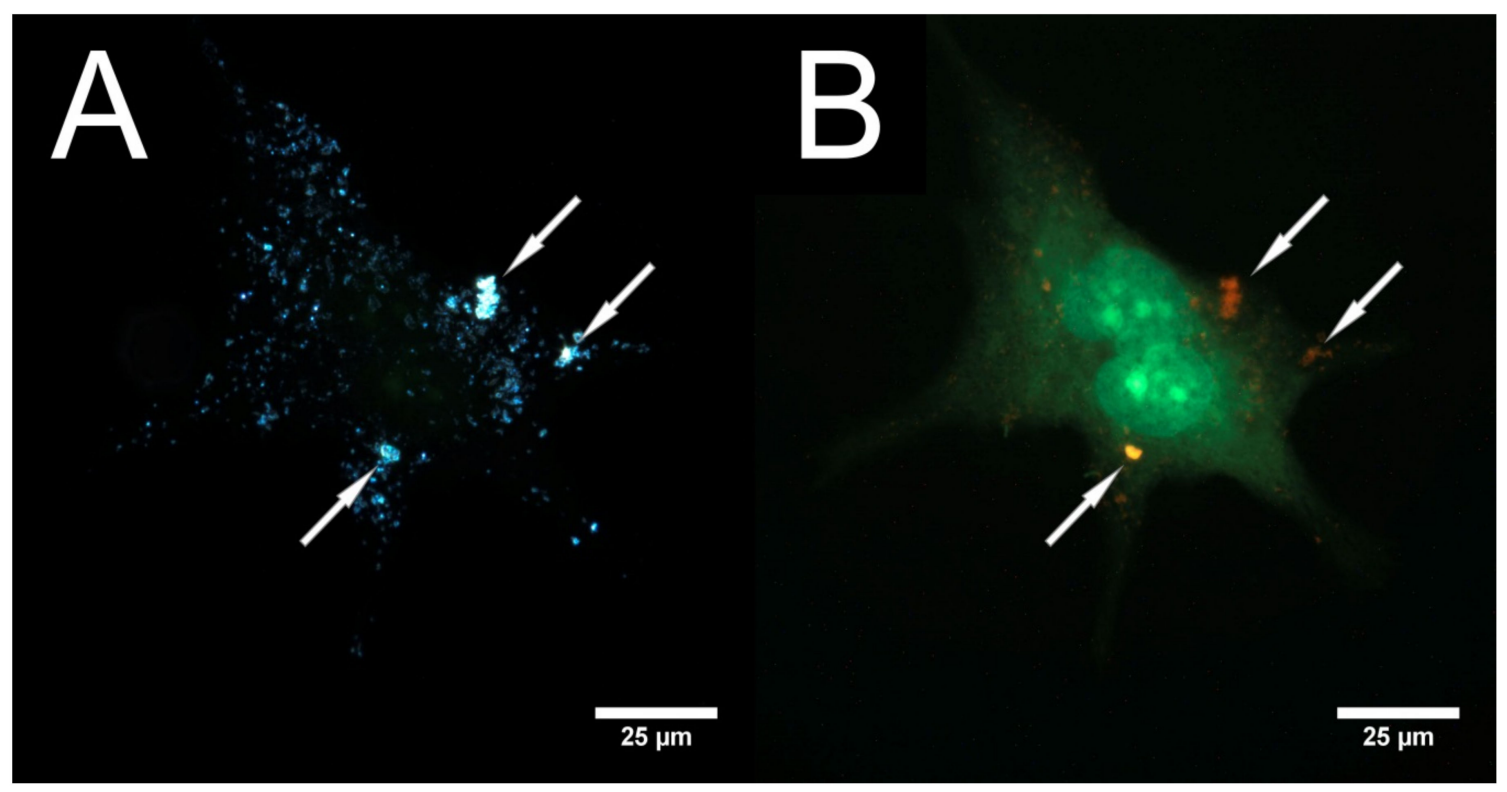
Visualization of PC-3 cell labeled with HNT-NH_2_-CdS: dark-field microscopy image of PC-3 with HNT-NH_2_-CdS (**A**) and fluorescence image of cells stained with ReadyProbes^®^ Cell Viability Imaging Kit (Blue/Green) (ThermoFisher) (**B**). HNT-NH_2_-CdS appeared as bright white spots (A, marked by arrows), or yellow and red spots (B), (marked by arrows).

More detailed distributions of the QD-nanotubes within the PC-3 cell are presented in Figure 4, demonstrating the correlative microscopy images taken using dark-field and epifluorescence microscopy. When observed using enhanced darkfield microscopy (Figure 4A), halloysite nanotubes appear as bright spots due to their good light-scattering properties. The cell membrane and cytoplasm can also be seen; however, at the same illumination intensity, the halloysite-lacking regions of the cell appeared to be faint. The corresponding fluorescence image of the same cell (Figure 4B) was stained with ReadyProbes^®^ viability dye, while HNT-NH_2_-CdS appeared as red spots, and their locations correspond to the brightest spots in Figure 4A, allowing us to correlate the QD fluorescence with halloysite light scattering.

### 3.3. Luminescence Stability

**Figure 5:**
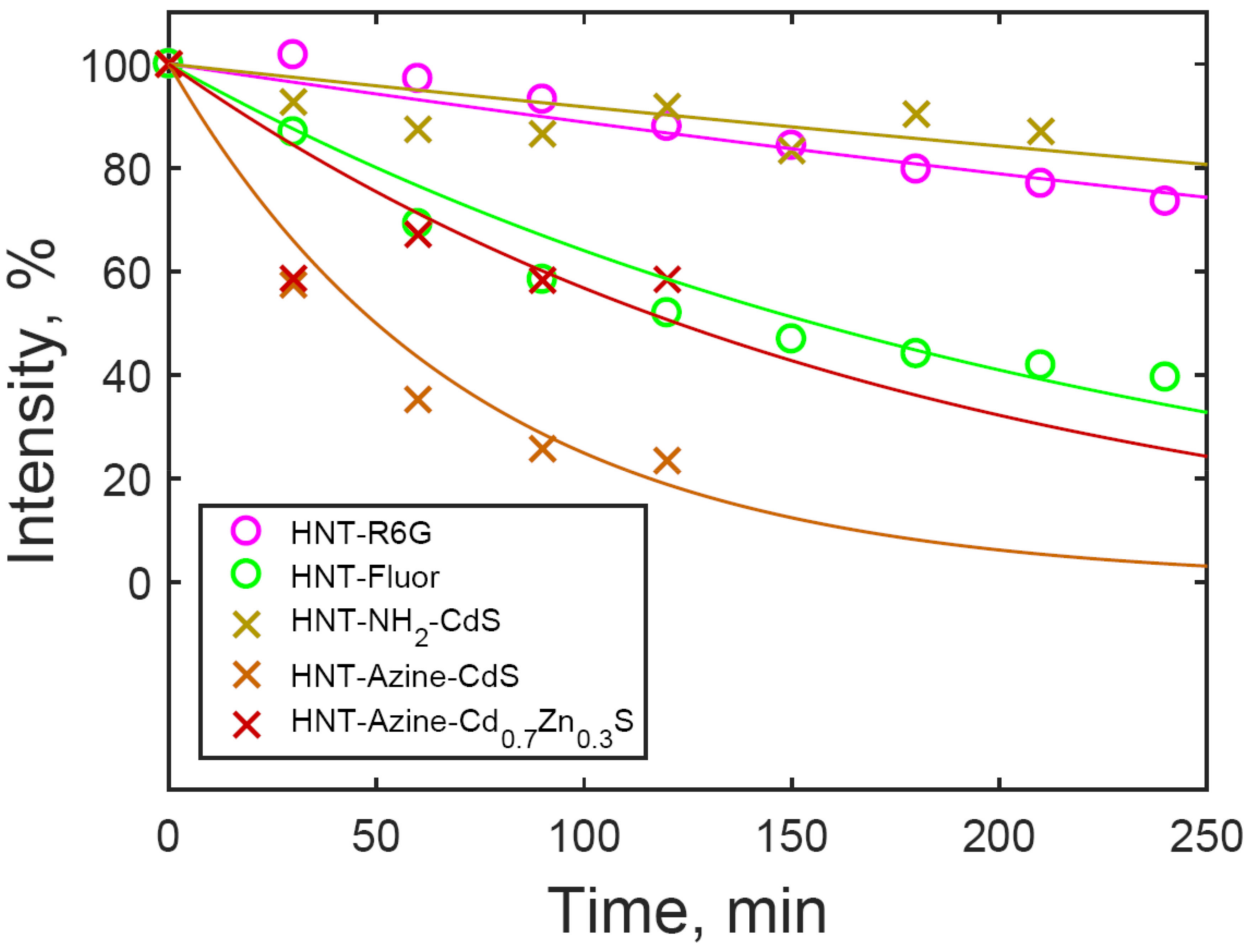
Time-dependent luminescence intensity of the synthesized materials (lines are first-order kinetics fits; see also Table S2 in Supplementary Materials).

Time-dependent change of the intensity of QD fluorescence was observed using a laser confocal microscope equipped with a 405-nm diode laser in time-series mode. The signal intensity was recorded every 30 min for 4 h, and is shown in Figure 5. In Figure 5, the photostability of synthesized materials is shown, along with the photostability of fluorescent dyes rhodamine 6G and fluorescein immobilized onto the halloysite (HNT-R6G and HNT-Fluor, respectively). It is known that photobleaching of fluorescent dyes follows pseudo-first-order kinetics [33,34], so experimental points (circles and crosses in Figure 5) were approximated by first-order kinetics fits (lines in Figure 5). The highest luminescence signal stability was observed for the HNTs-NH_2_-CdS (*k* = 0.00086 min^−1^), which is comparable with the photostability of rhodamine 6G (*k* = 0.0012 min^−1^). Azine-grafted materials (HNT-Azine-CdS and HNT-Azine-Cd_0.7_Zn_0.3_S) and halloysite-fluorescein composite (HNT-Fluor) showed a decreasing fluorescence intensity with time (*k* = 0.0139, 0.0057, and 0.0045 min^−1^, respectively).

### 3.4. Cytotoxicity

**Figure 6:**
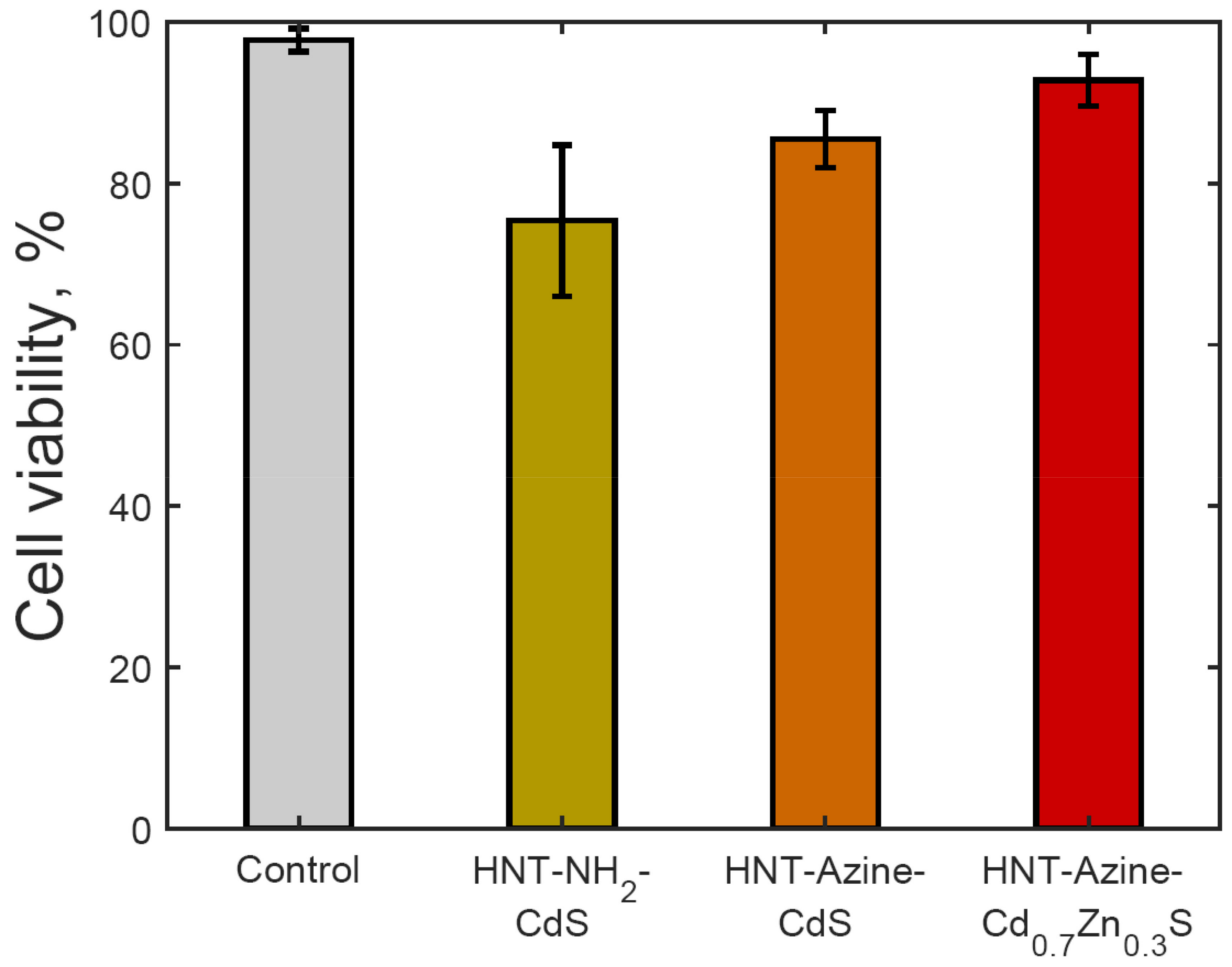
Flow cytometry data on viability of PC-3 cells exposed to halloysite-QD composites. Error bars are sample standard deviations (see also Figures S5–S8 in Supplementary Materials).

Earlier, we have shown the lack of toxic effects of halloysite-QD on cells with colorimetric assays and flow cytometry [20]. The toxicity of quantum dots depends on their size, chemical structure, and coating used [35,36]. We developed QDs that have a wide range of fluorescence, which is crucial for in vivo visualization. The toxicity of quantum dots was measured using the flow cytometry method. As one can see, viability of the cell is high in all cases as compared to the control (Figure 6). The percentage of live cells is higher when the HNT-Azine-Cd_0.7_Zn_0.3_S was used in cell cultivation.

## 4. Discussion

The synthesis of halloysite with grafted nitrogen-containing electron donating groups leads to the formation of fluorescent core-shell materials, which are comprised of QDs immobilized onto the surface of the clay nanotubes. The nature of the grafted groups affects the particle organization, size distribution, and spectral properties of the formed QD systems. Generally, azine-grafted materials provide a higher surface coverage of halloysite nanotubes with QDs and a narrower cadmium-containing nanoparticle size distribution. The immobilization of QDs onto the halloysite nanotubes prevents the aggregation of QD and ensures their good dispersibility in water. This immobilization approach is suitable even for nanoparticles without hydrophilic ligands or an amphiphilic polymer coating employed for the surfactant-stabilized nanoparticles [37].

One of the known problems of QD bioimaging applications is their blinking behavior, namely the transition between a photoluminescent “on” state and the Auger-recombination “off” state [38]. The halloysite-QD composites with high surface QD coverage provide a simple and robust workaround of the blinking problem because the luminescence is provided by many QD, thus making the blinking by individual QD negligible.

The produced halloysite-QD tubular nanocomposites demonstrate the affinity towards living cells; PC-3 cells were contrasted by the designed nanosystems, and no free fluorescent particles were detected. The remarkable differences in the fluorescence signal stability show that better particle size distribution and higher concentration of QD do not guarantee the stability of composites. HNTs-NH_2_-CdS formulations show higher photobleaching resistance, probably because of more sparsely distributed particles that are resistant to aggregation. The high photobleaching stability is crucial for real-life applications, such as study of the dynamics of intracellular processes, or prolonged diagnostics of organism malfunctions.

As one can see in Figure 3 and Figure 4, halloysite-QD composites are either taken up by human cells or adsorbed onto the cell membranes. The underlying mechanism is not clear yet, but based on earlier observations [39], we assume that uptake of halloysite occurs via endocytosis, and depends on proliferation rate of different cell cultures.

The surface modification of halloysite affects not only the synthesis and immobilization of cadmium chalcogenide nanoparticles, but also the cytotoxicity of the halloysite-QD composites. The HNTs-NH_2_-CdS formulations were most resistant to photobleaching, but at the same time demonstrated the highest cytotoxicity. Cadmium-containing QD are known to be cytotoxic because of Cd^2+^ ion emissions and direct interaction of QD with cell surface [40]. Thus, the immobilization of QD onto the surface of halloysite nanotubes may lower the cytotoxicity induced by the latter mechanism. Interestingly, among the azine-grafted halloysite composites, HNTs-Azine-Cd_0.7_Zn_0.3_S showed the lowest cytotoxicity together with moderate photobleaching resistance. The lower cytotoxicity of HNTs-Azine-Cd_0.7_Zn_0.3_S is probably due to the lower emission of Cd^2+^ ions from mixed cadmium-zinc sulfide, combined with the immobilization of QD.

We conclude that cadmium-zinc sulfide QD azine-grafted onto the halloysite clay nanotubes are the most promising materials for bioimaging.

## Supplementary Materials

The following materials are available online at http://www.mdpi.com/2079-4991/8/6/391/s1. Figure S1: Source TEM image of pristine HNT (Figure 1A); Figure S2: Source TEM image of HNT-NH_2_-CdS (Figure 1B); Figure S3: Source TEM image of HNT-Azine-CdS (Figure 1C); Figure S4: Source TEM image of HNT-Azine-Cd_0.7_Zn_0.3_S (Figure 1D); Figure S5: Flow cytometry graphs of the control sample of PC-3 cells; Figure S6: Flow cytometry graphs of PC-3 cells exposed to the HNTs-NH_2_-CdS sample; Figure S7: Flow cytometry graphs of PC-3 cells exposed to the HNT-Azine-CdS sample; Figure S8: Flow cytometry graphs of PC-3 cells exposed to the HNTs-Azine-Cd_0.7_Zn_0.3_S sample; Table S1: Measured diameters of electron-dense particles found in TEM images; Table S2: Photostability data for the synthesized nanomaterials.
