# Supplementary material for: Fluorescence and Cytotoxicity of Cadmium Sulfide Quantum Dots Stabilized on Clay Nanotubes
Source: Nanomaterials (Basel). 2018 May 31;8(6):391. doi: 10.3390/nano8060391 (PMC6026934; doi:10.3390/nano8060391)
Supplement: Supplementary file 1 [file nanomaterials-08-00391-s001.pdf]

nanomaterials

Supplementary materials

Fluorescence and Cytotoxicity of Cadmium Sulfide Quantum Dots Stabilized on Clay Nanotubes

Anna V. Stavitskaya[1], Andrei A. Novikov[1,*], Mikhail S. Kotelev[1], Dmitry S. Kopitsyn[1], Elvira V. Rozhina[2], Ilnur R. Ishmukhametov[2], Rawil F. Fakhrullin[2], Evgenii V. Ivanov[1], Yuri M. Lvov[1,3,*], and Vladimir A. Vinokurov[1]

[1] Functional Aluminosilicate Nanomaterials Lab, Gubkin University, Moscow, Russian Federation; novikov.a@gubkin.ru
[2] Bionanotechnology Lab, Institute of Fundamental Medicine and Biology, Kazan Federal University, Kazan, Republic of Tatarstan, Russian Federation; kazanbio@gmail.com
[3] Institute for Micromanufacturing, Louisiana Tech University, Ruston, USA; ylvov@latech.edu
* Correspondence: novikov.a@gubkin.ru; ylvov@latech.edu Tel.: +7-499-507-8692

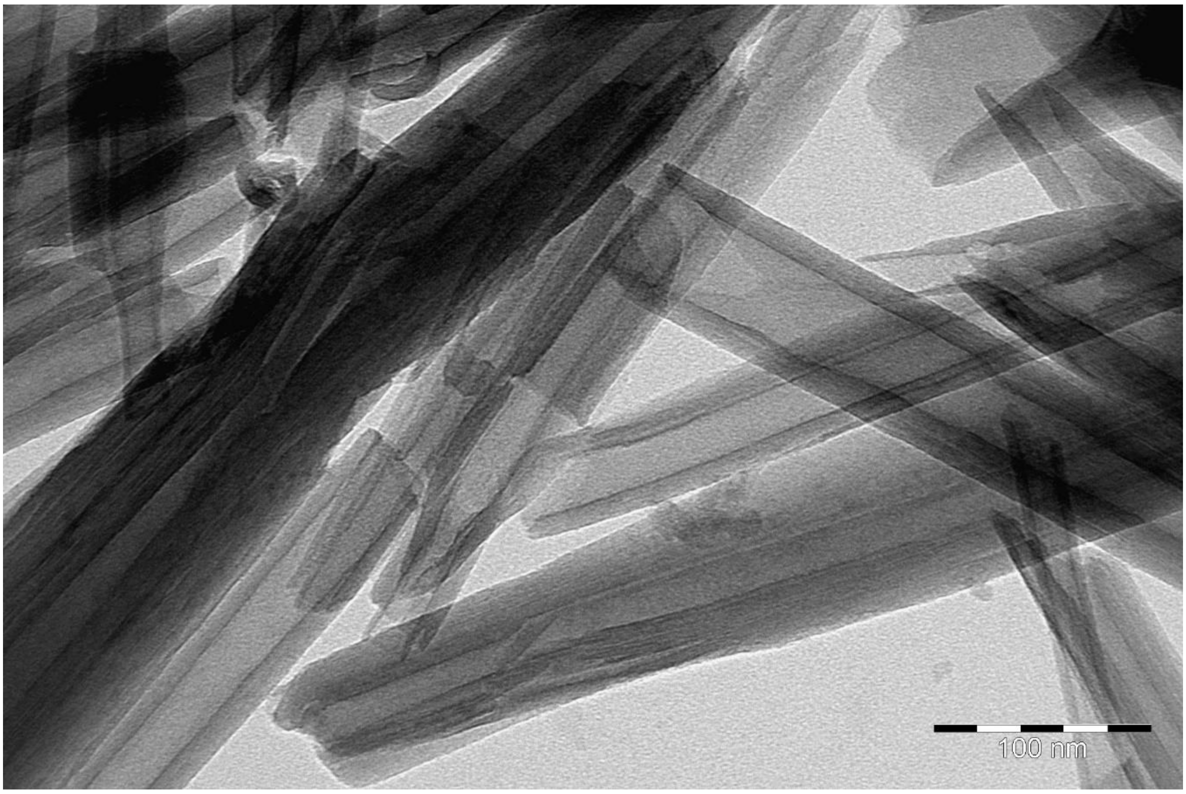

Figure S1. Source TEM image of pristine HNT (Fig.1A).

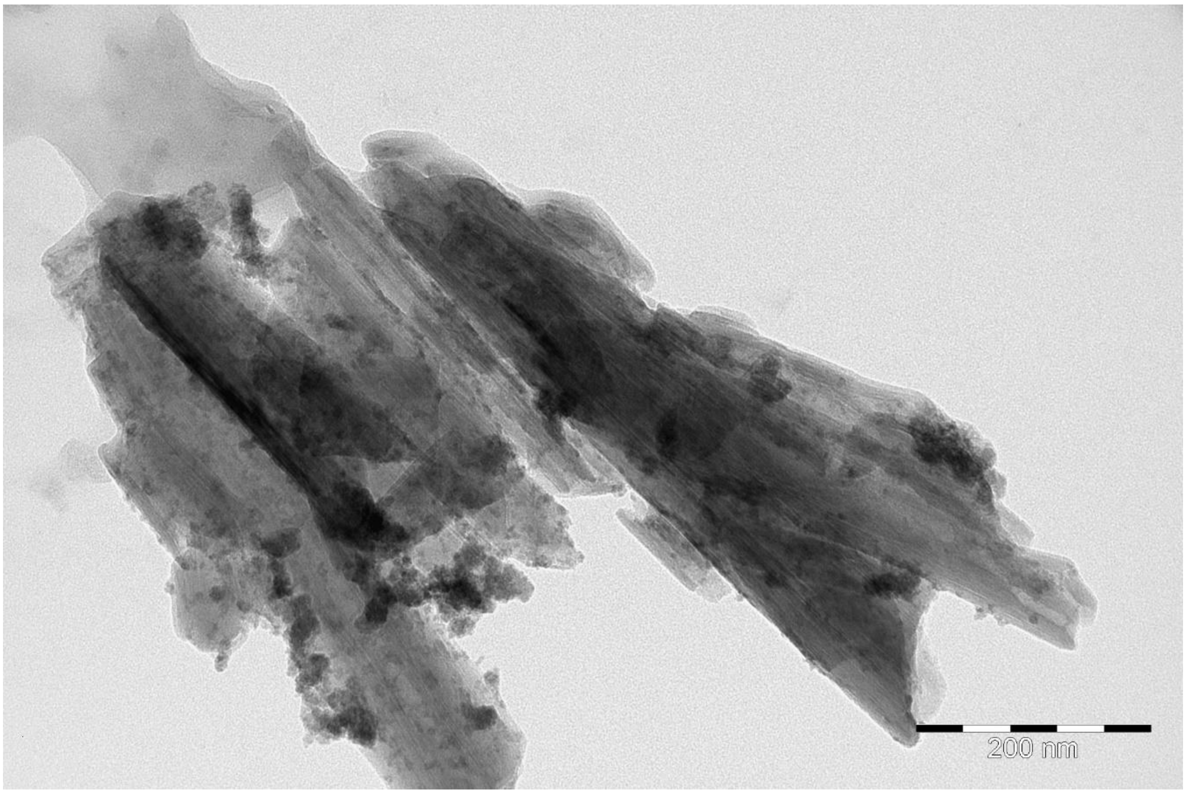

Figure S2. Source TEM image of HNT-NH_2-CdS (Fig.1B).

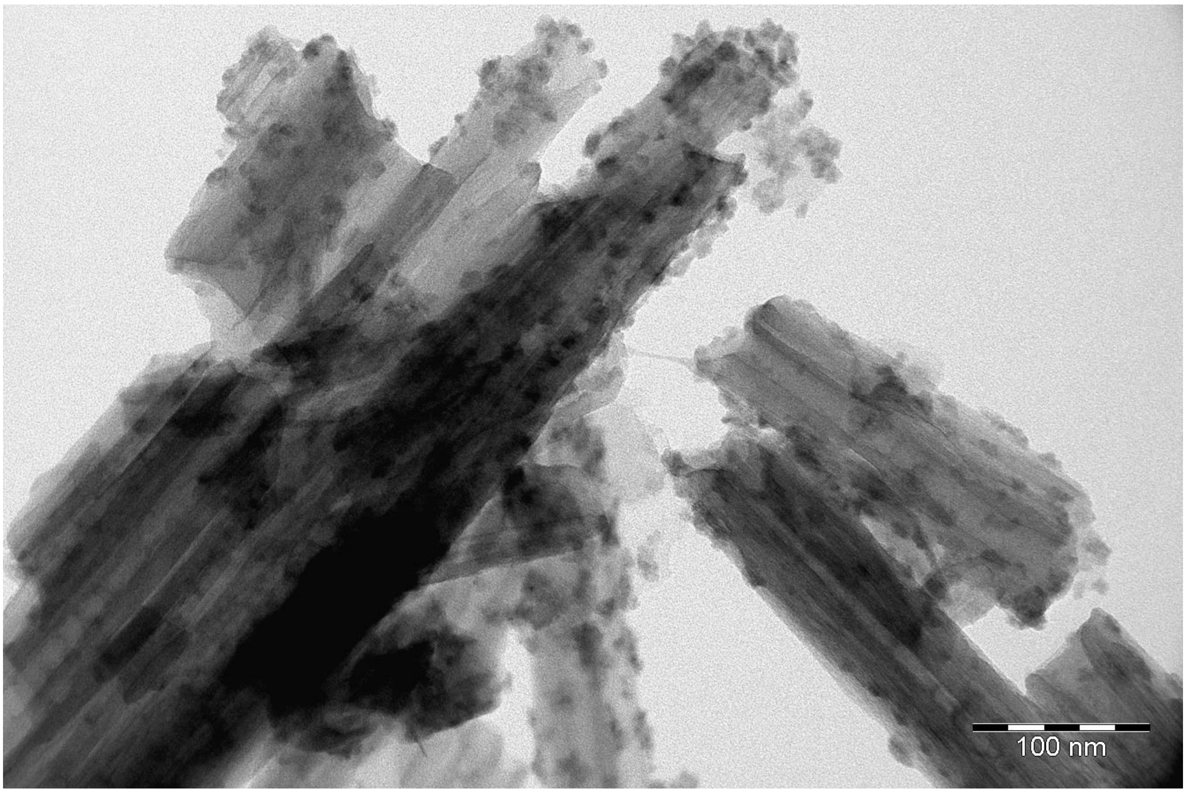

Figure S3. Source TEM image of HNT-Azine-CdS (Fig.1C).

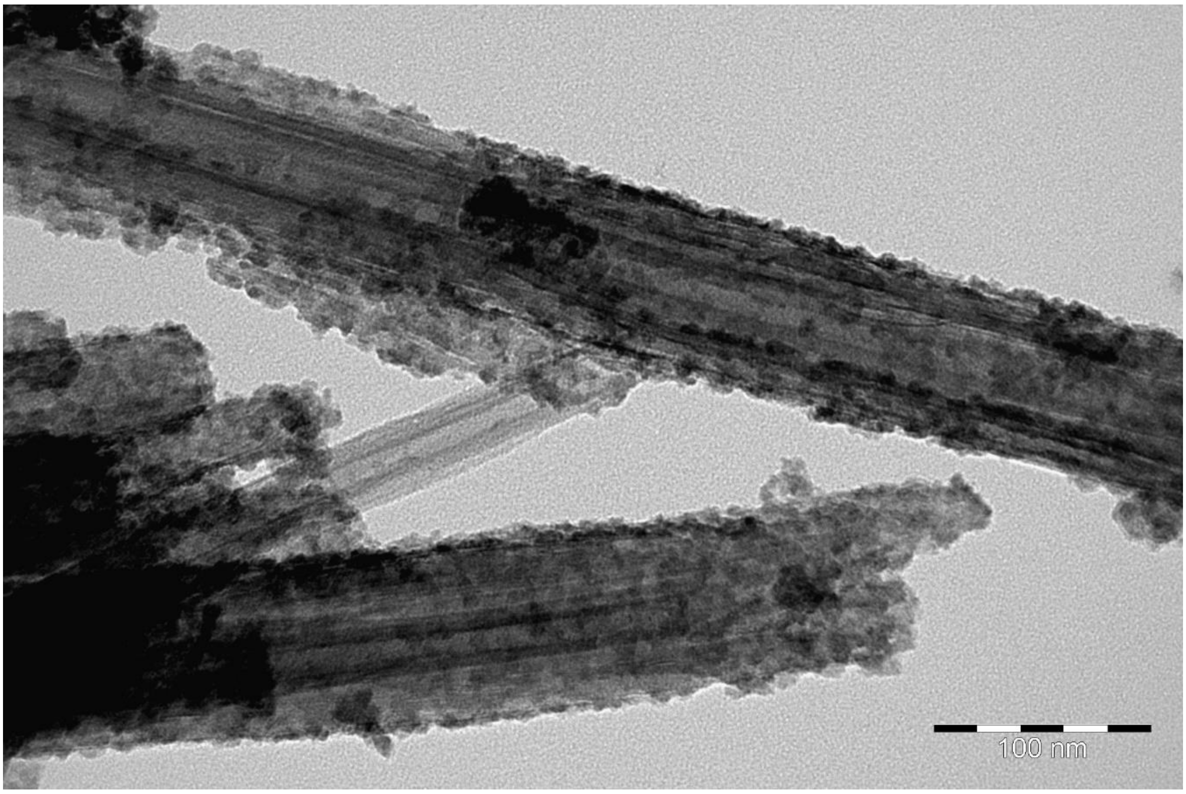

Figure S4. Source TEM image of HNT-Azine-$Cd_{0.7}Zn_{0.3}S$ (Fig.1D).

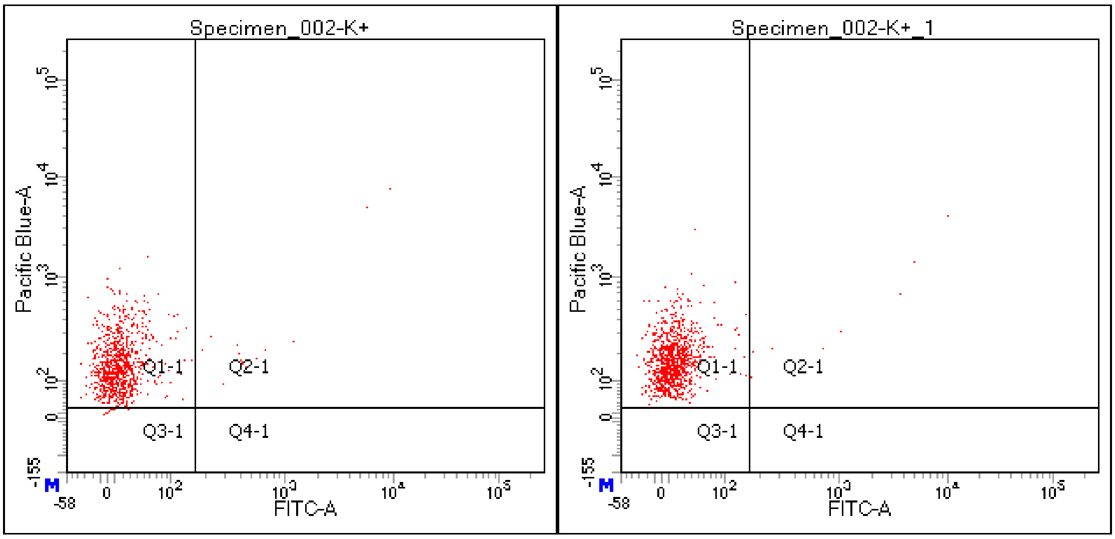

Figure S5. Flow cytometry graphs of the control sample of PC-3 cells (percentage of live cells shown in Figure 6 was calculated as Live,% = Q1-1 / (Q1-1 + Q2-1 + Q3-1 + Q4-1)×100%).

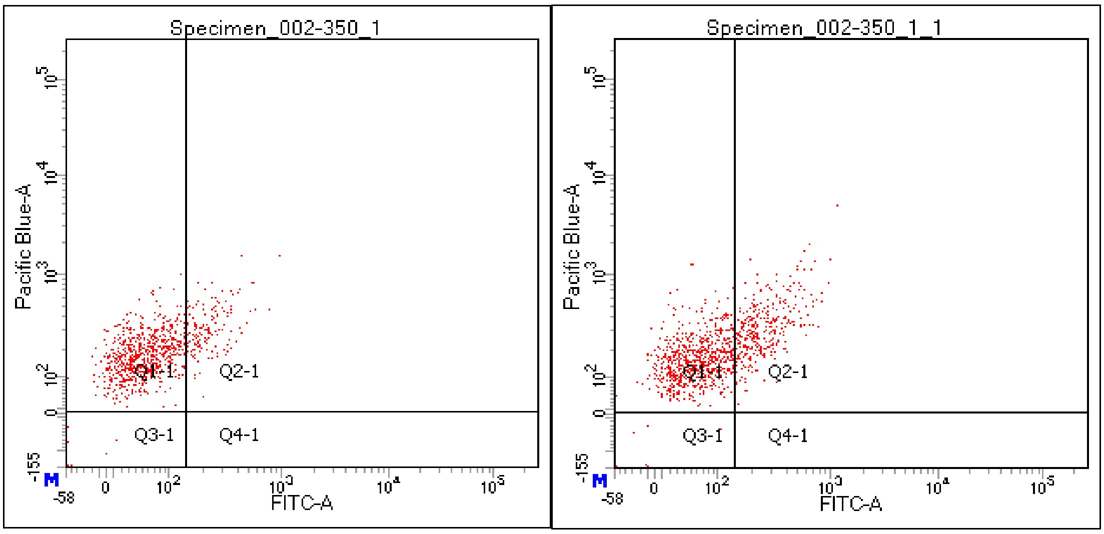

Figure S6. Flow cytometry graphs of PC-3 cells exposed to the HNTs-NH_2-CdS sample.

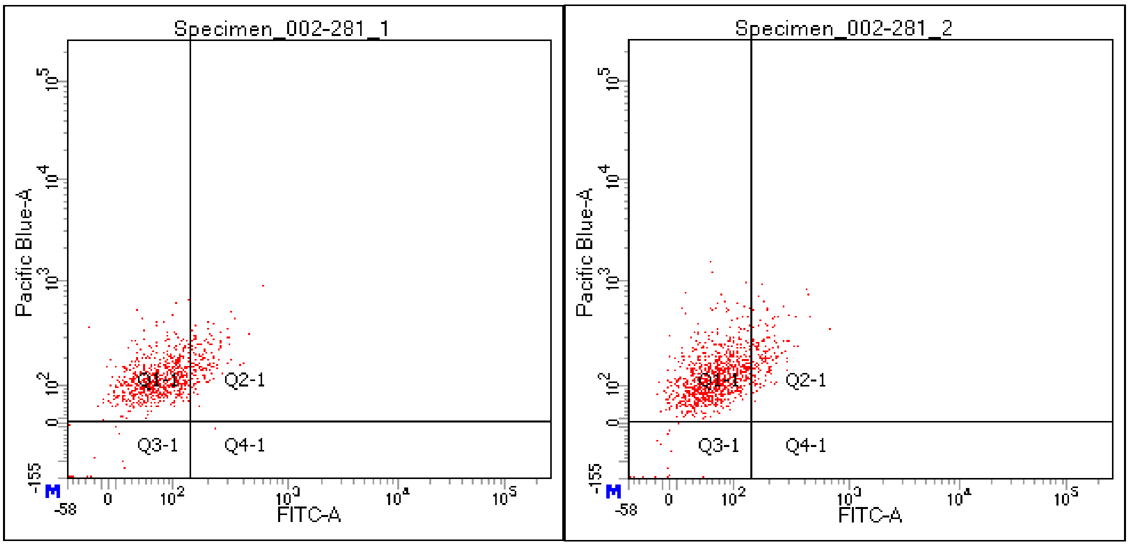

Figure S7. Flow cytometry graphs of PC-3 cells exposed to the HNT-Azine-CdS sample.

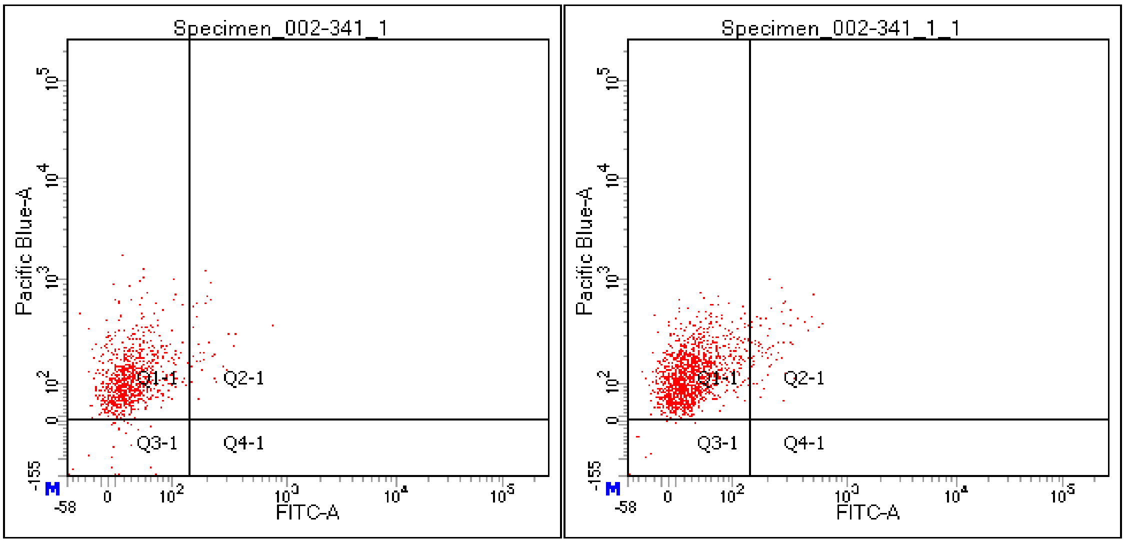

Figure S8. Flow cytometry graphs of PC-3 cells exposed to the HNTs-Azine-$Cd_{0,7}Zn_{0,3}S$ sample.

Table S1. Measured diameters of electron-dense particles found in TEM images.

#	Diameter, nm		
	HNT-NH_2-CdS	HNT-Azine-CdS	HNT-Azine-$Cd_{0.7}Zn_{0.3}S$
1	3.90	9.64	8.49
2	4.64	8.08	6.53
3	3.67	9.14	10.75
4	4.06	7.55	7.42
5	3.35	7.09	7.49
6	3.68	7.44	8.89
7	3.33	10.01	7.75
8	2.67	7.63	10.50
9	2.33	7.95	9.44
10	2.67	7.32	9.61
11	3.00	12.76	8.18
12	5.74	7.41	7.01
13	4.81	9.78	8.56
14	5.70	8.51	7.89
15	4.35	7.09	7.42
16	4.63	6.37	6.04
17	3.80	8.72	6.48
18	3.73	9.82	8.27
19	2.33	9.91	7.80
20	3.73	7.54	7.22
21	3.80	10.00	8.63
22	4.38	8.79	8.29
23	5.18	8.71	7.22
24	4.22	6.49	6.26
25	3.54	5.88	6.85
26	5.59	7.00	5.88
27	3.90	10.08	6.71
28	4.35	7.21	5.33
29	3.35	8.84	7.34
30	12.02	5.94	8.06
31	6.00	7.64	8.21
32	4.06	6.30	5.65
33	4.53	7.58	6.63
34	6.01	8.61	6.76
35	6.33	10.20	6.07
36	6.67	10.33	6.92
37	5.59	9.43	5.54
38	4.96	9.15	7.35
39	5.00	7.29	6.69
40	6.33	8.17	6.26
41	4.68	7.68	8.45

42	4.35	7.26	8.38
43	4.64	9.57	7.97
44	5.43	10.52	6.94
45	5.33	7.25	6.62
46	5.52	6.16	5.50
47	7.03	6.58	8.18
48	4.53	5.52	5.60
49	5.19	7.27	7.10
50	4.71	7.70	6.03
51	3.90	10.46	6.71
52	4.45	7.73	7.64
53	4.18	9.59	7.44
54	4.01	8.19	7.60
55	4.45	7.19	7.19
56	4.64	6.02	6.79
57	5.19	7.87	9.65
58	4.68	8.39	7.98
59	6.01	10.14	4.62
60	6.37	9.71	6.02
61	5.34	8.49	8.02
62	4.00	6.97	6.14
63	4.22	8.84	7.15
64	6.80	9.25	7.35
65	6.00	6.35	5.78
66	5.18	7.23	6.18
67	5.67	6.41	4.48
68	6.55	6.00	4.69
69	5.91	6.70	7.42
70	6.01	7.05	5.78
71	4.12	8.35	7.89
72	8.34	7.38	7.75
73	7.49	8.51	7.87
74	7.52	7.75	5.77
75	7.78	7.92	7.65
76	6.87	8.55	6.99
77	7.28	8.10	5.17
78	7.13	7.15	6.53
79	6.15	6.64	6.00
80	9.30	5.89	5.09
81	6.37	7.60	6.20
82	6.23	6.21	5.86
83	7.18	6.30	5.19
84	5.68	6.05	5.61
85	8.33	7.63	5.38
86	5.27	6.89	6.59

87	7.62	7.03	8.04
88	4.33	7.18	5.45
89	5.68	11.50	6.71
90	6.67	9.02	7.69
91	5.00	7.66	6.07
92	7.00	7.61	6.53
93	6.34	6.94	6.59
94	5.68	6.19	6.53
95	4.68	7.79	6.37
96	5.38	10.26	6.63
97	5.50	9.67	5.76
98	5.04	10.90	8.74
99	5.33	6.92	7.65
100	4.47	8.31	6.08

Table S2. Photostability data for the synthesized nanomaterials.

**Time, min**	**Intensity, %**				
	HNT-R6G	HNT-Fluor	HNT-NH_2-CdS	HNT-Azine-CdS	HNT-Azine-$Cd_{0.7}Zn_{0.3}S$
0	100.00	100.00	100.00	100.00	100.00
30	101.75	86.86	92.66	57.39	58.60
60	97.17	69.14	87.39	35.23	67.12
90	93.16	58.38	86.51	25.71	58.26
120	87.93	51.93	91.78	23.42	58.41
150	84.26	46.88	83.29		
180	79.68	44.04	90.35		
210	76.92	41.84	86.99		
240	73.46	39.51			

Time, min	log(I_0/I)				
	HNT-R6G	HNT-Fluor	HNT-NH_2-CdS	HNT-Azine-CdS	HNT-Azine-$Cd_{0.7}Zn_{0.3}S$
0	0.0000	0.0000	0.0000	0.0000	0.0000
30	-0.0174	0.1409	0.0762	0.5552	0.5344
60	0.0287	0.3691	0.1348	1.0432	0.3987
90	0.0708	0.5382	0.1449	1.3581	0.5402
120	0.1287	0.6553	0.0858	1.4514	0.5376
150	0.1713	0.7575	0.1829		
180	0.2272	0.8200	0.1015		
210	0.2624	0.8713	0.1394		
240	0.3084	0.9287			
k, min^{-1}	0.0012	0.0045	0.00086	0.0139	0.0057

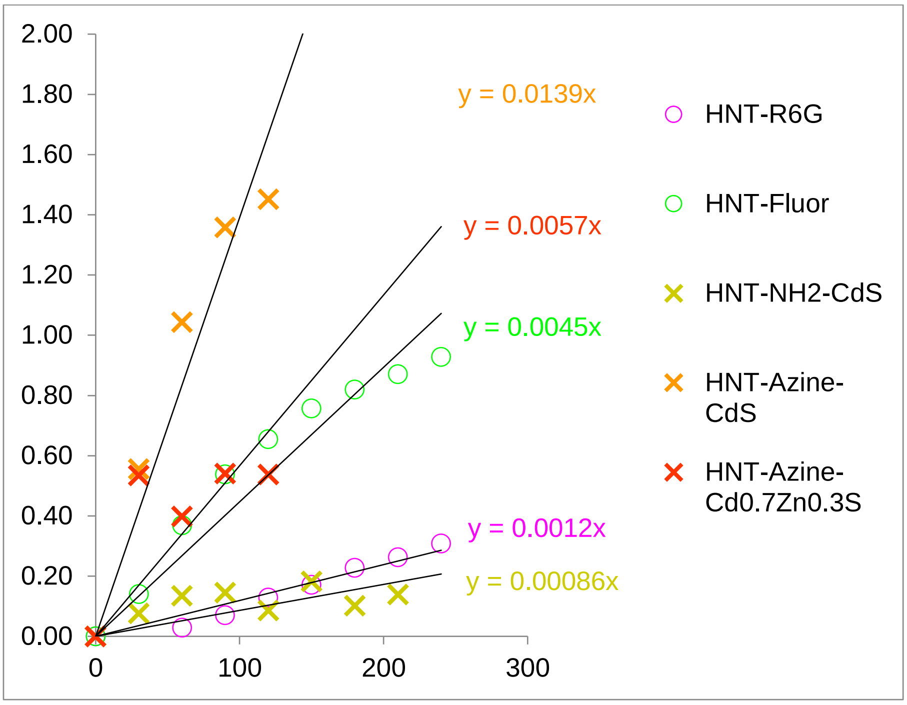
2.00
1.80
1.60
1.40
1.20
1.00
0.80
0.60
0.40
0.20
0.00
0
100
200
300
y = 0.0139x
y = 0.0057x
y = 0.0045x
y = 0.0012x
y = 0.00086x
HNT-R6G
HNT-Fluor
HNT-NH2-CdS
HNT-Azine-CdS
HNT-Azine-Cd0.7Zn0.3S
